# Co‐evolution of cerebral and cerebellar expansion in cetaceans

**DOI:** 10.1111/jeb.13539

**Published:** 2019-09-26

**Authors:** Amandine Sophie Muller, Stephen Hugh Montgomery

**Affiliations:** ^1^ Department of Zoology University of Cambridge Cambridge UK; ^2^ School of Biological Sciences University of Bristol Bristol UK

**Keywords:** brain evolution, cerebellum, cerebrum, mysticetes, odontocetes

## Abstract

Cetaceans possess brains that rank among the largest to have ever evolved, either in terms of absolute mass or relative to body size. Cetaceans have evolved these huge brains under relatively unique environmental conditions, making them a fascinating case study to investigate the constraints and selection pressures that shape how brains evolve. Indeed, cetaceans have some unusual neuroanatomical features, including a thin but highly folded cerebrum with low cortical neuron density, as well as many structural adaptations associated with acoustic communication. Previous reports also suggest that at least some cetaceans have an expanded cerebellum, a brain structure with wide‐ranging functions in adaptive filtering of sensory information, the control of motor actions, and cognition. Here, we report that, relative to the size of the rest of the brain, both the cerebrum and cerebellum are dramatically enlarged in cetaceans and show evidence of co‐evolution, a pattern of brain evolution that is convergent with primates. However, we also highlight several branches where cortico‐cerebellar co‐evolution may be partially decoupled, suggesting these structures can respond to independent selection pressures. Across cetaceans, we find no evidence of a simple linear relationship between either cerebrum and cerebellum size and the complexity of social ecology or acoustic communication, but do find evidence that their expansion may be associated with dietary breadth. In addition, our results suggest that major increases in both cerebrum and cerebellum size occurred early in cetacean evolution, prior to the origin of the major extant clades, and predate the evolution of echolocation.

## Graphical abstract

We show cetacean brain expansion is driven by increases in cerebrum (CX) and cerebellum (CB) size, two brain regions with functional and physical connections that are also expanded in primates. Brain images courtesy of the University of Wisconsin and Michigan State Comparative Mammalian Brain Collections (http://neurosciencelibrary.org)

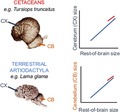

## 1 INTRODUCTION

Cetaceans are a remarkably diverse order, varying in size from <40 kg to 140 t (Montgomery, 2017; Nowak, 1999), but sharing a suite of derived adaptations that enable these ancestrally terrestrial mammals to occupy complex social and ecological niches in an obligatory aquatic environment. One such trait that has attracted particular attention, but remains relatively poorly understood, is a massively expanded brain. Cetaceans include species with the largest brain masses to have ever evolved (Ridgway & Hanson, 2014), and, until the emergence of the genus *Homo*, the most encephalized lineages on earth (Montgomery et al., 2013). The convergent trajectories of brain expansion in cetaceans and primates, and their possible behavioural and cognitive significance, have therefore garnered substantial interest (Marino, 1998; Marino et al., 2007).

However, differences in mammalian brain size can mask meaningful variation in brain structure and cellular composition (Barton & Harvey, 2000; Mota & Herculano‐Houzel, 2014). Cetacean brains are thought to have numerous features that deviate from general mammalian trends, including a thin and highly laminated cortex, extreme gyrification, low neuron density but high synaptic density, unique neuronal cell types, and small hippocampi that lack adult neurogenesis (Breathnach, 1960; Butti et al., 2015; Eriksen & Pakkenberg, 2007; Haug, 1987; Huggenberger, 2008; Marino, 2002, 2007; Morgane, Glezer, & Jacobs, 1990; Oelschläger & Oelschläger, 2009; Patzke et al., 2013; Poth, Fung, Güntürkün, Ridgway, & Oelschläger, 2005). Cetaceans also show a high degree of variation in several neural traits, including cerebellar size and cerebral cytoarchitecture (Hof & Van Der Gucht, 2007; Marino, Rilling, Lin, & Ridgway, 2000; Ridgway, Carlin, & Alstyne, 2018; Ridgway, Carlin, Alstyne, Hanson, & Tarpley, 2017; Ridgway & Hanson, 2014).

These derived and variable neural traits make cetacean brains an informative case study in understanding the constraints acting on brain structure. Brains are structured as networks of functionally specialized, but highly integrated and interdependent, components. Their functional properties depend on both the specialized tasks of specific brain regions and their integration. Hence, the degree to which brains are able to evolve in a modular, or “mosaic,” manner has been a major, long‐running debate in evolutionary neurobiology. One prominent model of brain evolution argues that developmental coupling between brain structures limits the degree to which brain composition can vary, but that these constraints ensure the functional integrity of the system is maintained as brains vary in size (Finlay & Darlington, 1995; Finlay, Darlington, & Nicastro, 2001). This “concerted” model is supported by apparent consistency in scaling relationships between the size of individual brain structures and total brain size across large phylogenetic distances (Finlay & Darlington, 1995; Finlay et al., 2001; Yopak et al., 2010). However, it is challenged by a more adaptationalist model in which the development and evolution of different brain regions are at least partly independent, allowing selection to bring about adaptive changes in brain structure (Barton & Harvey, 2000; Harvey & Krebs, 1990). These adaptations are reflected by grade shifts in the scaling relationships of specific brain regions, which indicate selective expansion that is independent of total brain size (Barton & Harvey, 2000; Barton & Venditti, 2014; Hall, Street, & Healy, 2013; Krebs, Sherry, Healy, Perry, & Vaccarino, 1989; Sherry, Vaccarino, Buckenham, & Herz, 1989; Sukhum, Shen, & Carlson, 2018), and in evidence of co‐evolution between functionally related structures that persist after removing the confounding effects of total brain size (Barton & Harvey, 2000; Iwaniuk, Dean, & Nelson, 2004).

Although these models are not mutually exclusive, understanding the degree to which brain structure—and presumably therefore function—is limited by development is key to several evolutionary questions. In general terms, these questions are centred around how to interpret allometric scaling relationships (Gould, 1966; Huxley, 1932), and the historically important debate about the importance of developmental integration in channelling patterns of evolution (Arnold, 1992; Cheverud, 1996; Gould & Lewontin, 1979; Finlay & Darlington, 1995). In the specific case of brain evolution, it is essential for understanding how behavioural specializations are manifest in the brain, whether behavioural or cognitive adaptations are a product of whole‐network properties or changes in the activity of specific operations in restricted brain regions (Logan et al., 2018), and for identifying the extent to which the genetic architecture of brain structure is the product of selection to maintain scaling relationships (Montgomery, Mundy, & Barton, 2016). Finally, given the propensity for comparisons of whole brain size when testing hypotheses about the evolution of cognition (Benson‐Amram, Dantzer, Stricker, Swanson, & Holekamp, 2016; Deaner, Isler, Burkart, & Van Schaik, 2007; MacLean et al., 2014), it is critical to know whether or not these comparisons can assume relative homogeneity in brain structure across taxonomic scales, or whether they are confounded by structural variance.

If mosaic changes in brain structure are common, direct comparisons of brain size can be misleading. As such, the unique morphology of cetacean brains may complicate direct comparisons with terrestrial mammals, in particular primates, where there is interest in the convergent evolution of brain expansion and cognition (Marino, 2002; Marino et al., 2007). Understanding how the differential expansion of individual brain components contributed to overall increases in brain size in each lineage is therefore crucial for accurately interpreting the significance of the convergent evolution of large brain size. One key feature of brain expansion in primates is the co‐evolution and coordinated expansion of the cortico‐cerebellar network (Barton & Venditti, 2014; Montgomery, 2017; Smaers, Turner, Gómez‐Robles, & Sherwood, 2018; Smaers & Vanier, 2019; Whiting & Barton, 2003). Although these structures tend to co‐vary across mammals as part of a three‐way relationship with the diencephalon, there appears to be a stronger co‐evolutionary relationship between the cerebellum and neocortex in primates (Barton & Harvey, 2000). Evidence from a range of taxa that the evolutionary trajectories of components of this system can be decoupled (Barton & Venditti, 2014; Hall et al., 2013; Sukhum et al., 2018) strongly suggests that the persistent correlated evolution between them reflects an adaptive functional relationship.

In primates, the expansion of cortico‐cerebellar system is partly characterized by grade shifts in size, relative to the rest of the brain, that may be decoupled in time (Barton & Venditti, 2014; Miller, Barton, & Nunn, 2019; Weaver, 2005). This implies some independent specialization, in support of the mosaic model of brain evolution, but also suggests that some form of constraint, imposed by the functional integration of these structures, couples their evolution over phylogenetic timescales (Barton & Harvey, 2000; Montgomery et al., 2016; Whiting & Barton, 2003). Volumetrically, the neocortex is the biggest component of this system and has attracted by far the most attention from cognitive and evolutionary neuroscientists (for critiques of this bias, see Barton, 2012; Parvizi, 2009). In contrast, the cerebellum has received much less attention, despite housing the majority of neurons in the brain (Barton, 2012; Herculano‐Houzel, 2009). Mounting evidence suggests that the cerebellum plays an important role in the development of typical and pathological variation in human behaviour and cognition (e.g. reviewed in Sokolov, Miall, & Ivry, 2017), potentially through the propagation of shared patterns of activity during learnt behaviour (Wagner et al., 2019), as well as in the evolution of primate brain expansion and cognition (Barton, 2012; Barton & Venditti, 2014).

Given the accumulated evidence of cortico‐cerebellar co‐evolution and specialization in primates, a major question is whether or not the same pattern is observed during independent episodes of brain expansion, such as cetaceans. Published comparative data on cetacean brain structure have been limited but paint a complex picture of cerebellar evolution in particular. Several early studies suggested that cetaceans have dramatically enlarged cerebella, with mysticetes having larger cerebella compared to odontocetes as a percentage of total brain size (Breathnach, 1960; Pilleri & Gihr, 1970). Marino et al. (2000) also noted that relative cerebellum volume in two dolphins was significantly larger than any primate. Several further studies have, however, noted extreme levels of variation in cerebellum size across cetaceans, with some species having relatively small cerebella (Maseko, Spocter, Haagensen, & Manger, 2012; Ridgway & Hanson, 2014). Ridgway and Hanson (2014) have also mooted an apparent cetacean‐specific dissociation between the normally tight correlation between the cerebellum and cerebrum. Extracting general trends from this literature is therefore difficult, particularly given the relatively small number of species for which data were available.

Recently, Ridgway et al. (2017) provided a new dataset of cetacean brain structure, with separate data on cerebrum and cerebellar volumes. This dataset, the result of collections made over the course of 50 years, provides brain size data for 770 individuals, of which 67 have data on both cerebrum and cerebellum volumes. These individuals unevenly represent 18 species, which makes it by far the largest dataset available to date. Using these data, Ridgway et al. (2017, 2014) presented a wide‐ranging analysis of variation in brain size, structure and growth across cetaceans. Key findings include observations of highly variable brain sizes and structure between major taxonomic groups, substantial variation in cerebellar size, as a percentage of brain volume and relative to body mass, and a derived ontogeny in which prenatal brain growth is both rapid and extended (Ridgway et al., 2017, 2014). Together, these results suggest that the origin and radiation of cetaceans involved substantial shifts in the selection regimes that shape brain development and structure.

However, Ridgway et al. did not compare their dataset to other mammals or examine patterns of cerebrum and cerebellum variation relative to the rest of the brain, which may be a more appropriate allometric control. They also chose to weigh individual data points equally, regardless of the number of samples per species, and to analyse their data without phylogenetic correction. Here, we revisit their data and add complementary analyses that aim to address the following questions: (a) Compared to other mammals, are cetacean cerebrum and cerebellar sizes both generally expanded relative to the rest of the brain? (b) If so, do they show coordinated patterns of variation, providing evidence of cortico‐cerebellar co‐evolution in cetaceans? (c) Does coordinated expansion preclude independent evolution? And (d) When did these increases in size occur, and do they explain key shifts in brain size and behaviour?

## 2 MATERIALS AND METHODS

### 2.1 Phenotypic and phylogenetic data

We obtained data on cerebral cortex (CX), cerebellar (CB) and whole brain mass from Ridgway et al. (2017) for 18 cetacean species, calculating mean masses where data for multiple individuals were available. “Rest of brain size” (RoB) was calculated by subtracting CX and CB from total brain volume. One species, *Megaptera novaeangliae*, was subsequently excluded from the dataset as CX and CB equalled total brain mass, suggesting one or both included additional structures. Component volume data for CX, CB and RoB for an additional 124 terrestrial mammals were taken from Carlisle et al. (2017) and Stephan, Frahm, and Baron (1981). We excluded olfactory bulbs from RoB volumes because the olfactory system is absent or greatly reduced in odontocetes (Oelschäger & Oelschäger, 2008), which, when compared to other mammals, could give the appearance of reduced RoB volumes relative to CB or CX volume. In theory, this could lead to a false signature of increased relative CB and CX size in cetaceans. The olfactory neuropils are still present in mysticetes (Thewissen, George, Rosa, & Kishida, 2011) but the available data are limited, prohibiting their exclusion in these species. However, in mysticetes the olfactory bulbs are proportionally quite small (~0.13% brain volume; Thewissen et al., 2011) so we consider their influence to have a negligible effect on our analyses. Given the small scale of deviation from isometric scaling between brain mass and volume, relative to measurement error (Isler et al., 2008), we also assume mass and volume are equivalent. Body mass was taken from the same source, with additional data from Jones et al. (2009) where data were missing. All brain and body data are available in Table S1A.

**Figure 1:**
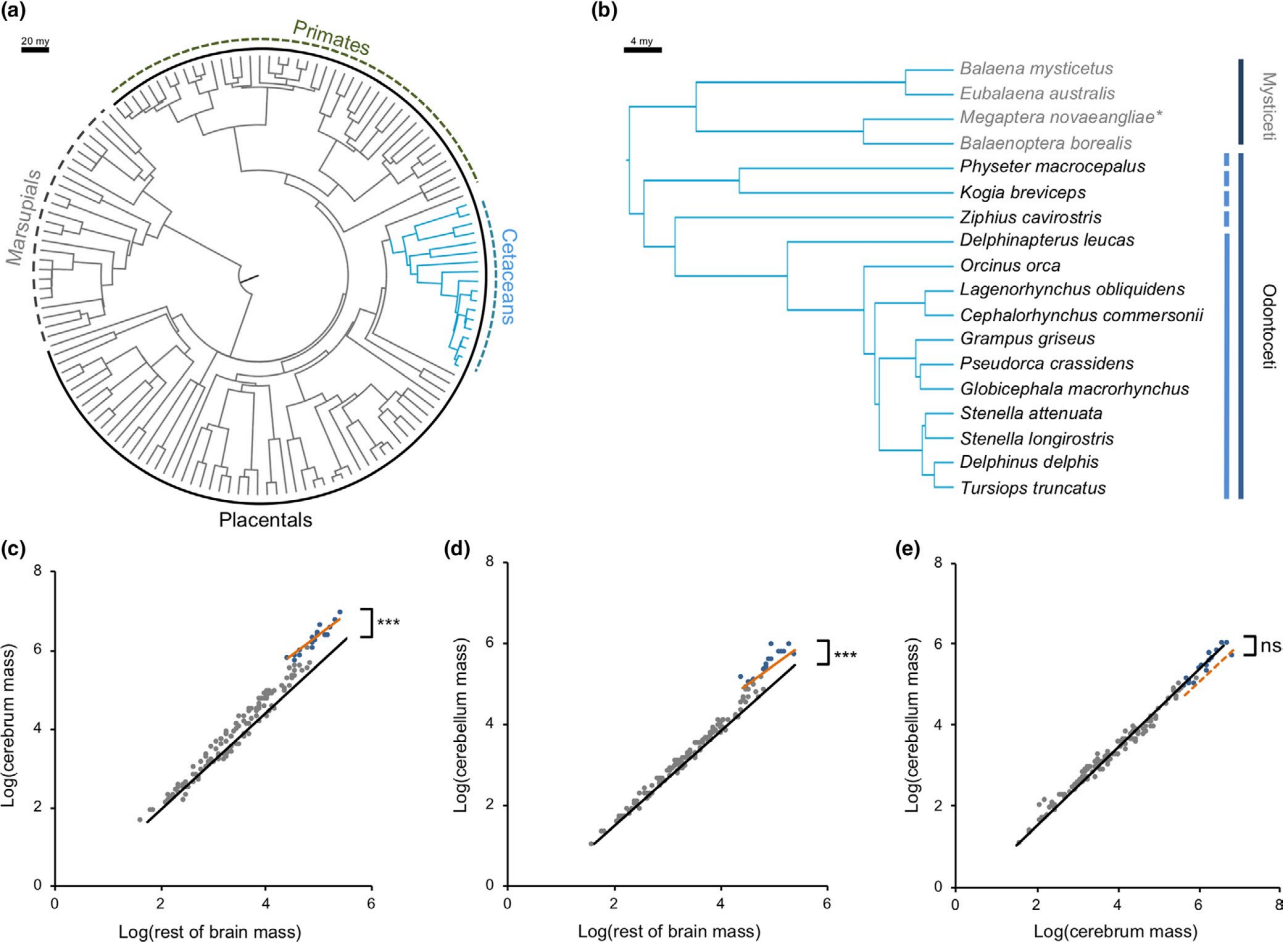
Phylogeny of species included in this study. (a) The all mammal dataset. Inner dashed line shows marsupial mammals, and inner solid line shows placental mammals. Outer green dashed line shows primates, and outer blue dashed line and branches show cetaceans. (b) Cetacean phylogeny, showing major taxonomic groups. Within odontocetes, the dashed/solid lines distinguish Delphinoidea from other odontocetes. Data for *Megaptera novaeangliae* (*) are available but were excluded as the sum of CB and CX equalled total brain volume, suggesting the inclusion of other components. (c, d) Log–log plots of scaling between (c) CX and RoB, (d) CB and RoB, and (e) CB and CX for all mammals (grey points/black line) and cetaceans (blue points/orange line). ***Significant grade shifts between cetaceans and other mammals at *p* < .001, ns indicates nonsignificant grade shifts

Phylogenetic trees for the included species were taken from two sources. For the analyses across mammals, we use the dated supertree produced by Bininda‐Emonds et al. (2007). However, the topology for cetaceans in this tree is poorly resolved. We therefore conducted cetacean‐only analyses using McGowen’s, Spaulding, and Gatesy (2009) dated phylogeny and spliced this tree into the mammalian supertree, re‐scaling branch lengths according to the ratio of divergence dates between the last common ancestor of Whippormorpha in the two trees (Figure 1a,b). Trees were visualized using FigTree v1.4.3 (http://tree.bio.ed.ac.uk/software/figtree/). The spliced nexus tree is provided in the Supporting Information.

### 2.2 Phylogenetic regressions

The core phylogenetic analyses were performed in BayesTraits (Meade & Pagel, 2016; available at http://www.evolution.rdg.ac.uk/BayesTraitsV3.0.1/BayesTraitsV3.0.1.html), using log_10_‐transformed species means. We first performed a series of phylogenetic *t‐*tests using phylogenetic generalized least squares (PGLS) in a maximum likelihood (ML) framework (Organ, Shedlock, Meade, Pagel, & Edwards, 2007) to examine variation in the size of each brain component between cetaceans and (i) all available terrestrial mammals, (ii) terrestrial placental mammals, (iii) just primates, and (iv) nonprimate placentals. This was repeated for CX and CB volume, including RoB volume as an independent variable to examine shifts in relative component size. A final mammal‐wide regression was performed to examine taxonomic differences in CX ~ CB scaling. For each ML analysis, we performed 1,000 iterations and ran the model with lambda, which measures phylogenetic signal, fixed to 1 and again with lambda freely estimated. The fit of these models was compared using a likelihood ratio test (Tables S2–S6). We examined CX ~ RoB, CB ~ RoB and CX ~ CB scaling within cetaceans using the same methods. In general, lambda was not significantly different from one and, where it was, it remained high. However, using PMC (Boettiger, Coop, & Ralph, 2012), we found that within cetaceans our power to accurately estimate lambda was reduced due to the smaller sample size, increasing uncertainty over the accuracy of these model comparisons (Supporting Information). As the results are consistent regardless of whether or not lambda is estimated freely, we report the full results for both sets of models in the Supporting Information, but focus on the models with lambda fixed to 1 in the main text.

In addition, we used phylogenetic mixed models implemented in MCMCglmm (Hadfield, 2010) to test whether results found within cetaceans are consistent when individual‐level data are used rather than species means. MCMCglmm controls for phylogenetic nonindependence by including a co‐variance matrix extracted from a given phylogenetic tree as a random factor in the model. All MCMCglmm analyses were performed using a Gaussian distribution with uninformative, parameter‐expanded priors for the random effect (G: V = 1,n *n* = 1, alpha.*n* = 0, alpha.V = 1,000; R: V = 1, *n* = 0.002) and default priors for the fixed effects. We report the posterior mean (P‐mean) of the cofactor included in each model and its 95% confidence intervals (CIs), and the probability that the parameter value is different to 0 (*P*_MCMC_).

### 2.3 Rate heterogeneity

We implemented the variable rates (VR) model in BayesTraits (Baker, Meade, Pagel, & Venditti, 2015; Venditti, Meade, & Pagel, 2011) to explore the distribution of rate heterogeneity in CX and CB evolution across the cetacean phylogeny. The VR model allows the rate parameter (*σ*) of a Brownian motion model to vary across individual branches or clades. A major advantage of this model is that it requires no *a priori* hypotheses about where rate shifts occur in a phylogeny and instead uses a Bayesian Markov chain Monte Carlo reversible‐jump procedure to optimize rate parameters across the tree (Baker et al., 2015; Venditti et al., 2011). This is suitable for our present analyses because we are interested about the presence of rate heterogeneity *per se*, and whether or not shifts in the rate of brain components are co‐incident, rather than in testing specific hypotheses about when or why these shifts occur.

We applied the VR model to CX and CB with RoB included as an independent variable in each case to permit an assessment of whether there is rate heterogeneity for CX and CB evolution after accounting for variation in RoB. We also performed an analysis with CX or CB included as the dependent variable in models with the other component included as an independent variable to confirm whether or not these traits can evolve independently. Due to the relatively small sample size, it is not possible to implement this model using only the cetacean dataset. The models were therefore run on the full mammal dataset, and the findings therefore apply to mammals in general and are not specific to cetaceans. However, evidence of rate heterogeneity within cetaceans can be inferred from the branch/clade‐specific scalars applied to branches within this order. The models were run for 100,000,000 iterations, sampling every 100,000 iterations after a burn in of 100,000,000 iterations. Marginal likelihoods (MLh) were calculated using the stepping‐stone sample, sampling every 100,000 iterations. Marginal likelihoods of the VR model were compared to the null model, in which σ cannot vary across the phylogeny, by calculating a log(Bayes Factor) (BF) as:

$$\text{BF}=2\left[\log\text{MLh}\left(\text{variable}\;\text{rates}\;\text{model}\right)-\log\text{MLh}\left(\text{null}\;\text{model}\right)\right]$$

BFs of 5–10 indicate “strong support” for the VR model, and BFs > 10 indicate “very strong” support. The VR logfile was processed using the online post‐processor tool (available at http://www.evolution.reading.ac.uk/VarRatesWebPP) to extract branch lengths scaled according to their mean/median rate of evolution. These were then plotted against raw branch lengths to highlight periods of high CX/CB evolution (Barton & Venditti, 2014). Linear regressions between sets of scaled branch lengths were performed in R (R Core Team, 2014) using the lm() function. Comparisons among models were performed using Akaike information criterion (AIC: calculated as (2 × number of parameters) − (2 × log[likelihood])) to identify the best supported model, where a lower value indicates a better fitting model, and a difference between models greater than two suggests a substantial difference (Burnham & Anderson,^2002^).

### 2.4 Ecological associations

Social complexity has long been seen as a potential explanation for brain expansion in cetaceans (Connor, Mann, Tyack, & Whitehead, 1998; Marino, 2002, 2007; Marino et al., 2007) and has recently been supported by an analysis of social repertoire size (Fox, Muthukrishna, & Shultz, 2017). As an initial test of whether social ecology is driving relative CX and/or CB expansion, we obtained data on social group and repertoire size from Fox et al. (2017) and performed a PGLS regression between CX and CB with each social trait, controlling for RoB size. We also repeated these analyses using diet breadth and latitude range (also from Fox et al., 2017) as a proxy for environmental heterogeneity, maximum dive time and two tonal traits, tonal range and tonal complexity (number of inflection points). Data on dive time were taken from Marino, Sol, Toren, and Lefebvre (2006), with additional and updated data from further studies (Argüelles et al., 2016; Barlow, Forney, Von, Saunder, & Urban‐Ramirez, 1997; Ishii et al., 2017; Krutzikowsky & Mate, 2000; Miller, Shapiro, & Deecke, 2010; Minamikawa, Watanabe, & Iwasaki, 2013; Silva et al., 2016). Tonal data were taken from May‐Collado, Agnarsson, and Wartzok (2007). All traits are continuous variables except for diet breadth which was coded by Fox et al. (2017) into four categorical groups. Data are presented in Table S1B. All analyses were performed using ML in BayesTraits with 1,000 iterations. The models were performed with lambda fixed to 1 and freely estimated (Table S6), but due to the relatively small sample size, we favour the more conservative models where lambda is fixed (see Supporting Information). All trait data have been deposited on Data Dryad (https://doi.org/10.5061/dryad.rm4368f).

## 3 RESULTS

### 3.1 Both the cerebrum and cerebellum are expanded in cetaceans

All brain components are larger in cetaceans than other mammals (CX: *t*_141_ = 3.853, *p* < .001; CB: *t*_141_ = 3.814, *p* < .001), but only narrowly so for RoB (*t*_141_ = 2.592, *p* = .042). The scaling relationship between the CX and RoB is significantly different in cetaceans compared to other mammals (*t*_141_ = 6.240, *p* < .001). This is also the case between CB and RoB (*t*_141_ = 5.749, *p* < .001). In both cases, the effect is a grade shift towards larger component volumes than predicted by the terrestrial mammalian scaling relationship with RoB (Figure 1c,d). However, the scaling relationship between CX and CB is consistent between cetaceans and terrestrial mammals (*t*_141_ = 0.549, *p* = .585; Figure 1e). The same results are obtained regardless of whether cetaceans are compared to all terrestrial mammals, only placental terrestrial mammals, only primates or only nonprimates (Tables S2 and S3).

### 3.2 The cerebrum and cerebellum co‐evolve in cetaceans, but exceptions occur

**Figure 2:**
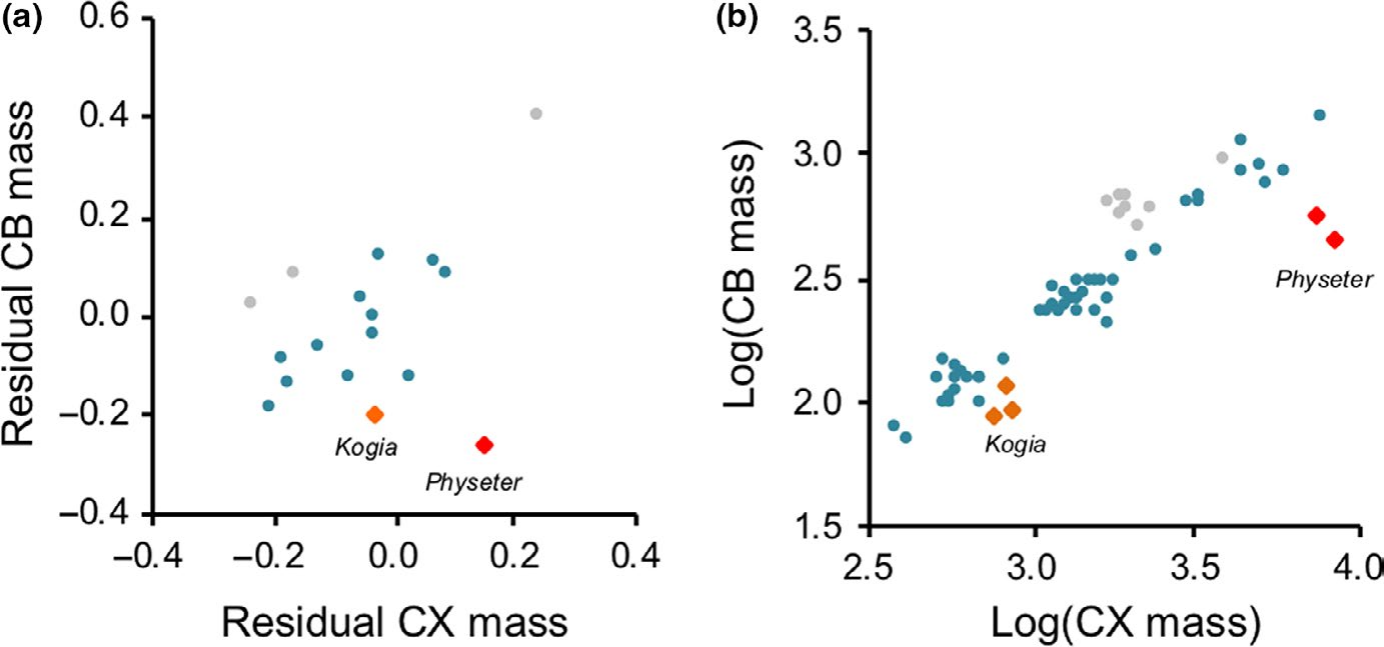
CB ~ CX co‐variance in cetaceans. (a) A plot of residual variance around a CB ~ RoB and CX ~ RoB regressions in cetaceans. Mysticetes are shown in grey, and odontocetes are shown in blue except for the two genera in Physeteroidea, *Kogia* and *Physeter*, which are shown as orange or red diamonds, respectively, to illustrate the position of *Physeter* as an outlier with the a smaller CB size than expected given CX/RoB size. (b) A plot of raw individual‐level data of CB ~ CX mass for all cetaceans, again highlighting the Physeteroidea to highlight consistency in the *Physeter* data

Consistent with the comparisons between cetaceans and terrestrial mammals, within cetaceans there is a significant association between CX and CB volume after correcting for RoB volume (*t*_13_ = 4.453, *p* < .001). We confirmed this result, which is based on species means, using all individual‐level data while controlling for species identity (P‐mean = 0.653, 95% CI: 0.446–0.834, pMCMC < 0.001). We also find a potential shift in this relationship between mysticetes and odontocetes (*t*_13_ = −3.749, *p* = .002; Figure 2a), although the data for mysticetes are very limited (*n* = 3) so this result should be revisited. To further explore these data, we calculated the residual variance around a regression between CX volume and RoB and plotted them against the residual variance around a regression between CB volume size and RoB. A nonphylogenetic regression between these phylogenetically corrected residuals is only significant when *Physeter macrocephalus* is removed (present *t*_15_ = 1.741, *p* = .102; removed *t*_14_ = 3.238, *p* = .006; Figure 2a), after which there is again a significant shift between suborders (*t*_12_ = −4.596, *p* < .001). Plotting the individual data also highlights the two *Physeter* individuals as outliers to the CB ~ CX scaling relationship (Figure 2b). This suggests that there is a potential deviation in CB ~ CX scaling between mysticetes and odontocetes and highlights specific lineages where the association between the expansion of both the CX and CB is relaxed, most notably in *P. macrocephalus* (Figure 2a). In contrast to previous studies (Ridgway et al., 2017), we do not find robust support for shifts in component scaling within odontocetes (Table S4); however, this analysis is again limited by sample size.

### 3.3 Rate heterogeneity in the evolution of cerebrum and cerebellum size

We next applied a variable rates (VR) model to both CX and CB, while controlling for RoB volume, using the full mammalian dataset. In both cases, the VR model was supported over a single‐rate Brownian motion model (CX, BF = 25.082; CB, BF = 19.489; Table S5), providing “very strong” evidence for significant variation in the evolutionary rate of both components that is independent of RoB volume, implying a degree of independent evolution between brain components. All variable rate models included branches within cetaceans that deviate from the background rate during mammalian evolution.

**Figure 3:**
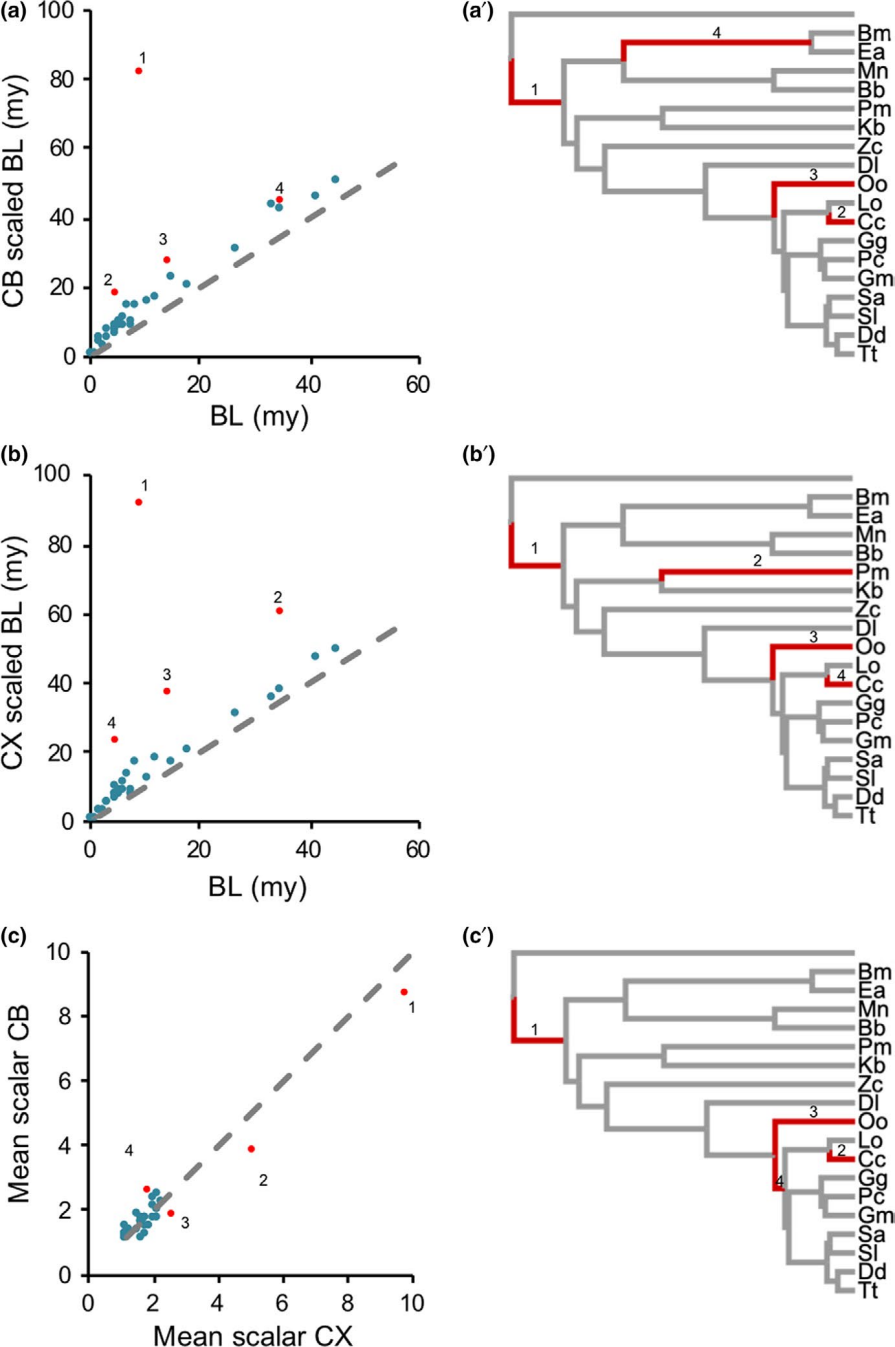
Scaled branch lengths from the variable rates models. (a) Scaled branch lengths against untransformed branch lengths from the variable rates model for CB, numbers indicate the top four branches with the highest deviation, which are coloured red and labelled in (a′). (b) Scaled branch lengths against untransformed branch lengths from the variable rates model for the CX, numbers indicate the top four branches with the highest deviation, which are coloured red and labelled in (b′). (c) Mean scalars from the variable rates model for CB and CX, controlling for RoB, in cetaceans. Numbers indicate the top four branches with the highest deviation, which are coloured red and labelled in (c′)

Focusing on cetaceans specifically, we plotted the *mean* scaled branch lengths against the untransformed branch lengths to visualize branches with an accelerated evolutionary rate (Figure 3a–c). The top four branches highlighted for the CB include the branch leading to the last common ancestor (LCA) of extant cetaceans, the terminal *Cephalorhynchus commersonii* and *Orcinus orca* branches, and the branch leading to the LCA of *Balaena mysticetus *and* Eubalaena australis* (Figure 3a,a′). For the CX, the branch leading to the LCA of extant cetaceans and the terminal branches of *P. macrocephalus*, *O. orca*, and *C. commersonii* are highlighted (Figure 3b,b′). However, the more conservative *median* scalars for both components only indicate deviation for two branches for both structures, the branch leading to LCA of extant cetaceans and the terminal *C. commersonii* branch.

We next repeated the variable rates test using CX mass while controlling for CB volume (and vice versa, where the results obtained were highly similar, Table S5). Again, the variable rate model was supported over a single‐rate Brownian motion model (BF = 28.635), suggesting that despite their tendency to co‐evolve, these components have also varied independently through time. Plotting the within‐cetacean *mean* scaled branch lengths for the CX and CB VR models highlights several branches with higher evolutionary rates for CX or CB (Figure 2c,c′). However, the *median* scalars only indicated deviation for the branch leading to LCA of extant cetaceans.

### 3.4 Expansion of the cerebrum and cerebellum both contribute to variation in brain size

**Figure 4:**
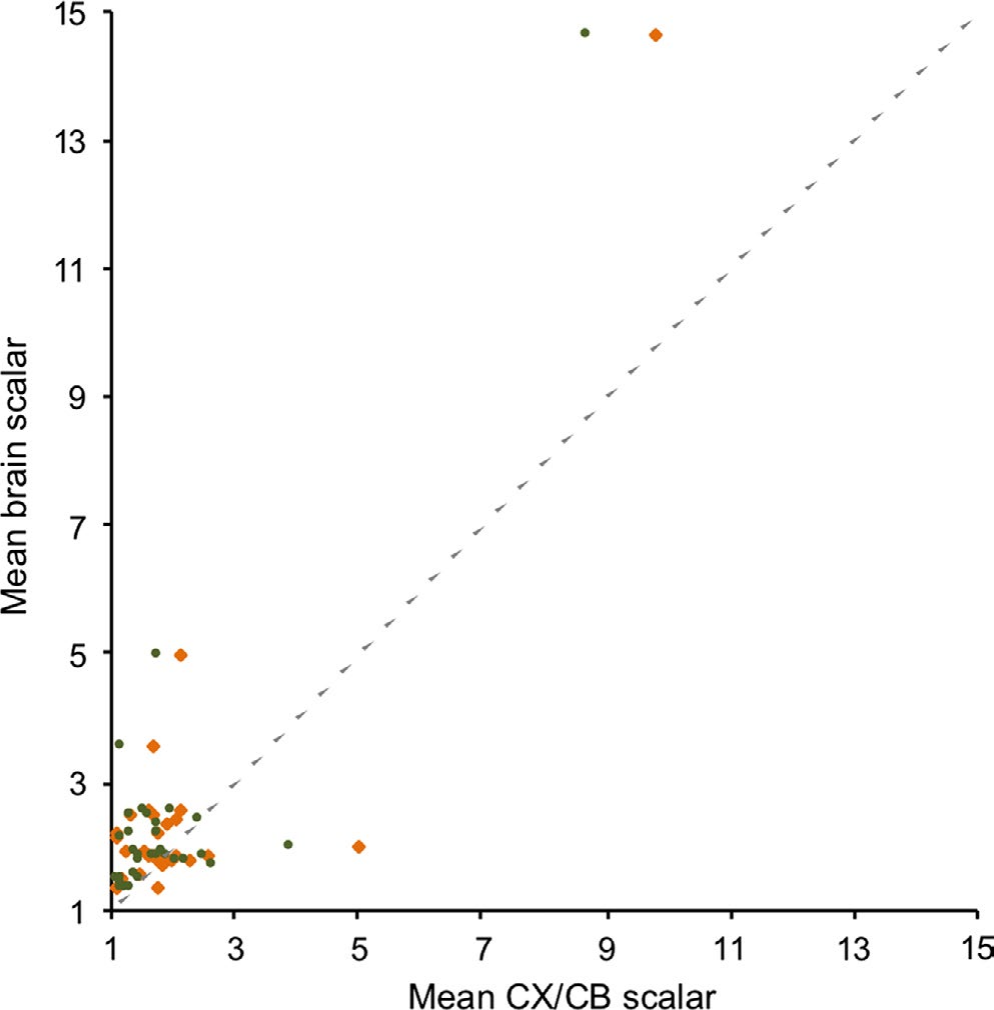
Mean scalars from the variable rates model for CB (orange) and CX (green), controlling for RoB, in cetaceans, plotted against the mean scalar for brain mass, controlling for body mass. The dashed line indicates a 1:1 relationship

To explore whether increases in relative CX or CB mass drive brain expansion in cetaceans, we repeated the VR analysis on brain size, while controlling for body mass, across all mammals. Again, the VR model is supported over a constant‐rate model (BF = 25.467) indicating significant rate heterogeneity in the evolution of mammalian brain size when correcting for body mass. Within cetaceans, the mean scalars of each branch (indicating variation in *σ*) for body corrected brain size are not significantly associated with the mean scalars for either CX (*t*_30_ = 1.208, *p* = .237) or CB (*t*_30_ = 1.0885, *p* = .287; Figure 4). However, this could reflect the dominant effect of body mass on variation in relative brain size in cetaceans (Montgomery et al., 2013). Indeed, across cetaceans the size of the CB (*t*_9_ = 18.853, *p* < .001) and CX (*t*_9_ = 98.363, *p* < .001) is significantly associated with whole brain size, after accounting for RoB volume, but body mass is not (*t*_9_ = 2.200, *p* = .055). Removing body mass from the model also significantly improves the fit (ΔAIC = 3.944). We take this to indicate that variation in the relative size of the CB and CX is associated with variation in whole brain size. However, a VR analysis of brain size, without controlling for body mass, does not support significant rate heterogeneity across mammals (BF = −0.326), precluding a reliable test of whether or not changes in CB, CX and whole brain size occur co‐incidentally in cetaceans.

### 3.5 A preliminary assessment of ecological traits driving cortical and cerebellar expansion

Finally, we explored the relationship between the relative size of both components and key ecological variables. We first focused on social ecology, which has often been invoked to explain cetacean brain expansion. We found no evidence of the predicted *positive* linear association between CX mass and either social repertoire size (*t*_13_ = −0.525, *p* = .608) or social group size (*t*_13_ = −1.734, *p* = .107), while controlling for RoB mass. This prediction is also not met for CB, where we find no association between CB mass and social repertoire size (*t*_13_ = 0.480, *p* = .639) and a weak *negative* association with social group size (*t*_13_ = −3.033, *p* = .010). Similar results were found when CX, CB and RoB were analysed in a single multiple regression (Table S5). In the latter case, there is a suggestion of an association between social group size and RoB (*t*_11_ = 2.594, *p* = .022). We repeated these analyses with whole brain and body mass and again found no significant association with either social trait (Table S6).

Finally, we also explored the relationship between CB and CX size and diet breadth, latitude range, maximum dive time and tonal complexity (Table S6). We found only one trait with evidence of an association between either brain component; both CB (*t*_13_ = 2.574, *p* = .023) and CX (*t*_13_ = 2.967, *p* = .011) show evidence of a positive association with diet breadth. These results are however vulnerable to correction for multiple tests and should be treated as preliminary. However, both associations were also present when the number of dietary categories was reduced to 3, as only one species in the original dataset was assigned to category 4 (CB *t*_13_ = 2.484, *p* = .027; CX *t*_13_ = 2.374, *p* = .034).

## 4 DISCUSSION

Compared to most other extant mammals, cetaceans have evolved under dramatically different selection regimes. Comparisons between cetacean brains and those of terrestrial mammals suggest that this included changes in the selection pressures and constraints that shape how brains evolve. Using the largest available dataset on cetacean brain components, together with comparable data from terrestrial mammals, we revisited key questions about cetacean brain evolution. Despite several unique features (highlighted above), we confirm that cetacean brain expansion shares a common dependency on cortico‐cerebellar expansion with terrestrial mammals, in particular primates (Barton & Harvey, 2000; Herculano‐houzel & Sherwood, 2010; Whiting & Barton, 2003). Within cetaceans, we find evidence of coordinated cortico‐cerebellar evolution at a phylogenetic scale (Maseko et al., 2012; Montgomery, 2017; Ridgway et al., 2017; Smaers et al., 2018), but also evidence that suggests the capacity for independent changes in the size of each component. We tested three common hypotheses that seek to explain the behavioural relevance of larger cerebrums or cerebella in cetaceans and provide preliminary evidence of an importance of diet breadth, a proxy of the diversity of prey types. Below, we discuss each of these results in further detail.

We found robust evidence that both the CB and CX are expanded in cetaceans relative to the rest of the brain, but also find a general pattern of co‐evolution between them. However, this phylogenetic co‐ordination appears to mask a more flexible relationship. This is indicated by significant rate heterogeneity in CX/CB volume across mammals, after accounting for their co‐variation with each other or with RoB, and by individual branches showing evidence of higher rates of change in one structure or the other. We interpret this pattern as indicating a combination of distinct and shared selection pressures acting on the CX and CB, with the presence of some form of functional constraint that limits the extent to which one structure can diverge without reciprocal changes in the other (see Montgomery et al., 2016, for further discussion). This functional dependence is consistent with known patterns of connectivity (Ramnani, 2006), coordinated activity (Wagner et al., 2019) and evidence from other mammals, particular humans, that the coordinated action of the cortico‐cerebellar system is important for many behaviours (Barton, 2012; Parvizi, 2009; Sokolov et al., 2017).

An alternative explanation for the apparent co‐evolution of brain components argues instead that the evolution of brain structure is constrained by shared developmental programs that couple component size to whole brain size (c.f. Finlay & Darlington, 1995; Finlay et al., 2001). One predicted pattern of a strictly concerted model of brain evolution is that structures that develop late in a neurogenic time course, such as the cerebrum and cerebellum, are more prone to disproportionate expansion (“late equals large” Finlay & Brodsky, 2006; Finlay et al., 2001). This hypothesis is strongly debated (Weisbecker, 2009) and nevertheless cannot explain our results as it argues that disproportionate expansion is caused by conserved allometric scaling across groups and hyperallometric scaling exponents. Our results instead provide two pieces of evidence that suggest that cetacean brain structure provides a clear counter example to a general prevalence of overarching developmental constraints on brain structure (c.f. Marino et al., 2000). First, major grade shifts are observed in the size of both the cerebrum and cerebellum relative to the rest of the brain; hence, their increase in relative size is not due to conserved hyperallometric scaling. Second, across mammals in general, and among cetaceans, there is further evidence of independent evolution of both structures. Our results are therefore consistent with a “mosaic” model of brain evolution (Barton & Harvey, 2000), and data from molecular studies in other vertebrates that suggest selection may act on independent sets of genes and developmental pathways that control the size of each brain component (e.g. Noreikiene et al., 2015; Harrison & Montgomery, 2017; Montgomery et al., 2016).

The question that follows, of course, is what is the behavioural relevance of these expanded brain regions? Here, we focused on three hypotheses that seek to explain at least some variance in overall brain size and test whether they explain variation in either relative cerebrum or cerebellum size. First, we sought to test whether variation in CB/CX size is explained by variation in social ecology. The social complexity of extant cetaceans is well recognized and includes evidence of cooperative behaviour, social transmission of behaviour, and dynamic social structures (Connor, 2007; Marino et al., 2007). Although the social complexity of odontocetes is often emphasised, many of these behaviours are also observed in mysticetes (Marino, 2007; Simmonds, 2006; Whitehead, 2011). Several authors have suggested increases in cetacean brain size could be explained by selection associated with social cognition (Connor et al., 1998; Marino, 2002; Shultz & Dunbar, 2010); however, evidential data have been limited. Recently, Fox et al. (2017) reported an association between cetacean group size, a composite measure of social repertoire size and brain size (absolute and body‐size corrected). We revisited these data to test whether or not group or social repertoire size has a simple linear relationship with CB or CX size, independently of RoB. We found no support for this hypothesis.

A major component of cetacean social ecology is acoustic communication. The importance of auditory information arguably further increased in odontocetes following the evolution of echolocation. Indeed, brain structure in cetaceans has clearly evolved to support perception and processing of auditory information (Marino, 2007; Marino et al., 2002; Ridgway, 2000). Cerebellar expansion is also shared among mammals with pronounced auditory adaptations, including echolocating bats and odontocetes, and elephants, which utilize long‐distance infrasonic vocalizations (Hanson, Grisham, Sheh, Annese, & Ridgway, 2013; Maseko et al., 2012; Paulin, 1993). Indeed, neural activity in the cerebellum has been linked to the processing of acoustic signals (Baumann & Mattingley, 2010; Jen & Schlegel, 1980; Singla, Dempsey, Warren, Enikolopov, & Sawtell, 2017) and is consistent with the role of this brain structure as an adaptive filter that tracks patterns of predicted and observed sensory input (Marino et al., 2002; Paulin, 1993; Ridgway, 2000). We therefore next explored whether vocal repertoire (measured as tonal range and tonal complexity; May‐Collado et al., 2007) was associated with CB or CX mass. Again, we found no significant association with either brain structure. Across social and tonal traits, the closest result to a nominal significance threshold of 0.05 was between RoB and group size, which could suggest a potential association between social behaviour and an expanded midbrain, which includes several auditory structures (Marino, 2007). However, this trend was weaker for tonal traits.

The third hypothesis we explored is that cetacean brain composition is largely shaped by foraging behaviour. When discussing the striking differences between *Orcinus* and *Physeter* cerebellar sizes, Ridgway and Hanson (2014) suggested that either reduced visual processing or prolonged periods of oxygen depletion during deep water diving might limit investment in *Physeter* cerebellar neuron number (see also Marino et al., 2006). Indeed, our analysis supports the contention that *Physeter* has a unique brain composition among cetaceans, with an expanded CX but relatively small CB (Figure 2). Although the data are limited, both individuals in our dataset are adults and have consistent brain compositions. Ridgway et al.’s (2017) original dataset also includes two further individuals with data for CB but not CX size, which are again consistent with the two individuals we include in our dataset. This suggests the small CB size observed for *Physeter* is unlikely to be due to sampling biases or measurement error. However, although it is possible that the constraints imposed by deep diving are particularly pronounced or limited to *Physeter*, we find no general association between maximum dive time and relative CB/CX mass. Finally, Fox et al. (2017) also reported an association between body‐size‐corrected brain mass and two measures of nonsocial ecological complexity, diet richness and geographic (latitudinal) range. While we found no evidence of an association between geographic range and RoB‐corrected CB or CX mass, we do find a significant association between both RoB‐corrected CB and CX size and a categorical measure of dietary breadth. We stress that these results should be viewed as preliminary because they are based on a relatively small dataset and we have performed tests for seven ecological traits. However, they are consistent with evidence that the behavioural challenges associated with foraging exert strong selection pressures on the evolution of brain size and structure (Clutton‐Brock & Harvey, 1980; Barton 1998; DeCasien, Williams, & Higham, 2017; Powell, Isler, & Barton, 2017; Fox et al., 2017). We therefore encourage further studies of the role of nonsocial cognitive specialisation in cetacean evolution.

We also acknowledge that, while we find no evidence that CB/CX expansion is driven by social ecology, our dataset (*n* = 17) is substantially smaller than Fox et al.’s (*n* = 46) and we do not replicate their findings with whole brain size using this subset of data. It is therefore possible that social traits do contribute to CB/CX expansion but we do not detect its effects for various potential reasons. First, it is possible that these null results merely reflect a combination of examining a relatively small phylogenetic dataset, and the use of behavioural data that is potentially highly “noisy,” particularly given the challenge of collecting these data for cetaceans. In part, the limitations of the data come from using proxy measures of cognition. For example, Fox et al. (2017) suggest there is a nonlinear relationship between group size and social complexity, and even when examining measures of social organization (aggregations/megaopods/mid‐sized associations), there is significant variation in social repertoire size, suggesting the full repertoire of social complexity is poorly captured. Similarly, May‐Collado et al.’s tonal data focus solely on tonal sounds but broadband, burst‐pulsed calls also play important roles in cetacean social communication (Lammers, Au, & Herzing, 2003; Sørensen et al., 2018) and may support social interactions between individuals of species that that do not produce tonal sounds, and which do not aggregate on the surface frequently enough to accurately record social complexity (Sørensen et al., 2018). A second issue is data coverage. Despite attempts to correct for biases in publication rates (Fox et al., 2017), the availability and quality of data are likely in part determined by a species’ ecology and may not fully represent biologically relevant variation in behavioural traits across cetaceans. Even in large, comprehensive datasets, variability in trait data from alternative sources can result in differing results in comparative analyses (Powell et al., 2017), and this problem is likely to be more pronounced in hard to study species.

It is also possible that our results are influenced by different selection pressures acting on CB/CX mass in different parts of the phylogeny, or reciprocally across time. Indeed, in both cetaceans and terrestrial mammals no single ecological trait appears to explain variation in relative brain size or structure (Barton, Purvis, & Harvey, 1995; DeCasien et al., 2017; Fox et al., 2017; Powell et al., 2017). In a small dataset, testing interdependencies between multiple traits is unreliable, making it hard to discern a full model of what drives the evolution of cetacean brain structure. However, given that by far the largest shift in evolutionary rate for both the CB and CX occurred on the branch leading to the last common ancestor of extant cetaceans, and that there is no pronounced shift at the origin of echolocation in odontocetes, it at least seems unlikely that CB/CX expansion was primarily driven by the evolution of echolocation, as has been previously suggested (Marino et al., 2000; Paulin, 1993; Ridgway, 2000; Ridgway & Hanson, 2014). Changes in the internal structure of the CB/CX that have been associated with echolocation (Marino et al., 2000) would therefore have evolved on the back of an already expanded cortico‐cerebellar system. A similar exaptation hypothesis has been proposed to explain how expansion of the cerebellum in apes could have initially supported increased fine motor and sequential learning needed for tool use, but was later co‐opted and adapted to support the evolution of language in hominins (Barton, 2012).

An early origin of an expanded CB is consistent with some endocasts of early archaeoceti (Edinger, 1955; Kellogg, 1936; but see Bajpal, Thewissen, & Sahni, 1996; Breathnach, 1955, 1960), suggesting the switch to an obligate aquatic lifestyle may have itself altered the selection regimes acting on the size of major brain components. Indeed, there is evidence of convergent changes in cerebellar morphology between cetaceans and pinnipeds, although these are also shared by hominoid primates (Smaers et al., 2018). Teasing apart which were the key selection pressures during this period is difficult, as the shift to an aquatic environment likely involved major changes in sensory processing and motor control, both of which have been suggested as drivers of variation in CB size (Marzban et al., 2011; Maseko et al., 2012; Ridgway & Hanson, 2014). We also note that likely changes in size‐related constraints on brain expansion that are associated with aquatic weightlessness, major increases in body mass (Huggenberger, 2008; Marino, 1998; Montgomery et al., 2013) and an energy‐rich diet (Evans et al., 2012) may have resulted in the unique brain structure and mode of expansion characteristic of cetaceans (Marino, 2004). Although CB structure is thought to be widely conserved (Larsell, 1967; Sultan & Glickstein, 2007), the low neuronal density, nonlaminar connectivity and “cortical adjacency” of the CX (Marino, 2002, 2007) could conceivably have downstream effects on CX‐CB connectivity and co‐evolution in cetaceans.

Understanding the interacting selection pressures that have produced the expanded brains of cetaceans remains a daunting challenge. Given the potential for brain components to evolve independently, and to reflect complex patterns of reciprocal dependencies on other brain regions and with multiple ecological traits, we suggest that efforts to identify simple relationships between crude traits like whole brain size and compound traits like general cognition will have limited success. Improved and more precise data for both neuroanatomical and behavioural traits are sorely needed, and the collections obtained by Ridgway et al. (2017) and others represent a major contribution towards this effort. Given the difficulty in obtaining comparative datasets, renewed long‐term efforts and increased academic cooperation will be required to provide robust behavioural data, access to cetacean brain samples and imaging data, as well as tissue samples suitable for genome and transcriptome sequencing.

## CONFLICT OF INTEREST

The authors declare no conflict of interest.

## Data Availability

Data available from the Dryad Digital Repository: https://doi.org/10.5061/dryad.rm4368f
