# Supplementary material for: Co‐evolution of cerebral and cerebellar expansion in cetaceans
Source: J Evol Biol. 2019 Sep 26;32(12):1418–31. doi: 10.1111/jeb.13539 (PMC6916408; doi:10.1111/jeb.13539)
Supplement: Supplementary file 1 [file JEB-32-1418-s001.docx]

Co-evolution of cerebral and cerebellar expansion in cetaceans

**Supplementary Information:**

- 1. Uncertainty in estimating lambda
  2. Power to estimate lambda
  3. **Uncertainty in estimating lambda**

For each component we estimated phylogenetic signal (lambda) under Maximum Likelihood (ML). Across mammals, cerebellum (CB), cerebrum (CX) and rest-of-brain (RoB) have lambda estimates that are not significantly different from 1 (Table S2). When RoB is included as a cofactor, lambda is significantly different from 1 (Table S2) but remains high (CB = 0.977; CX = 0.970). In contrast, estimating lambda in the cetacean only dataset produces ML estimates ranging from 0 (RoB) to 0.923 (CB). However, in the majority of cases the ML estimate is not significantly different from 0 or 1, suggesting a high degree of uncertainty in the parameter estimation. To explore this we used the Markov chain Monte Carlo (MCMC) approach implemented in BayesTraits to produce a posterior distribution of lambda estimates. The chains were run for 20,000,000 iterations with a burn-in of 10,000,000, and a sample period of 100,000. For both CB and CX the distributions of lambda estimates using the cetacean-only dataset are extremely broad indicating a high degree of uncertainty (Fig S1A-B, S2A-B)..In contrast, when the full mammal dataset is used there is a much narrower range of estimates (Fig S1C, S2C).

**
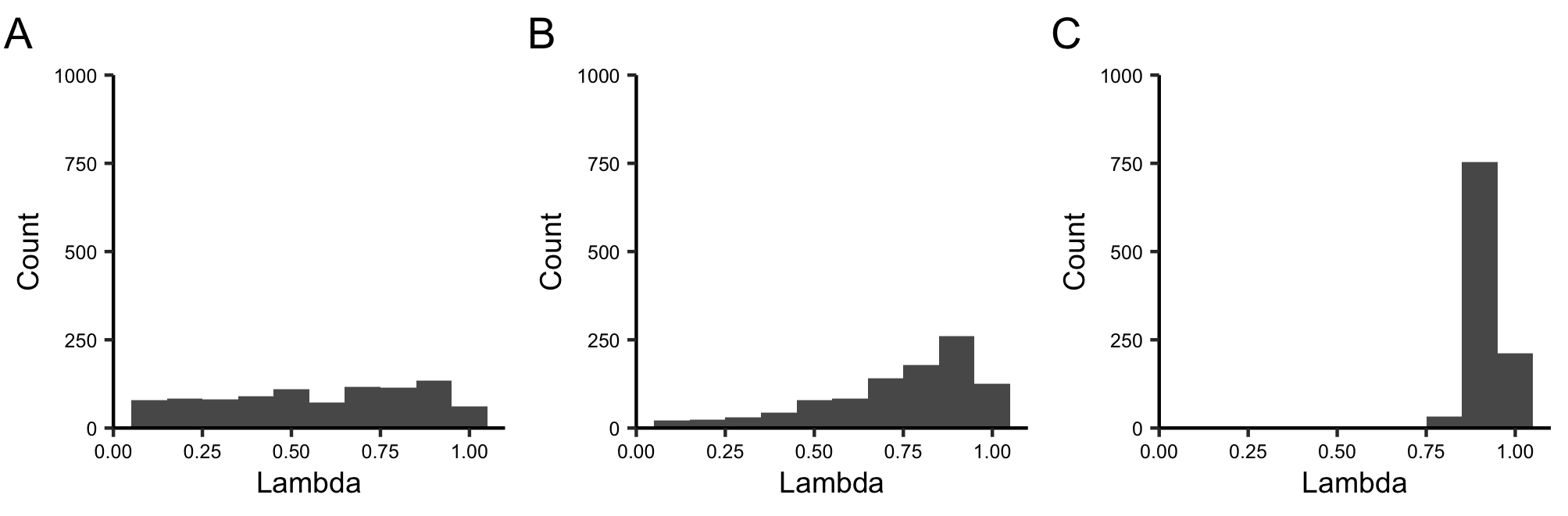
Figure S1:** Posterior distributions of lambda estimates for CB volume. A) cetaceans only; B) cetaceans only with RoB included as a cofactor; C) all mammals with RoB included as a cofactor.

**
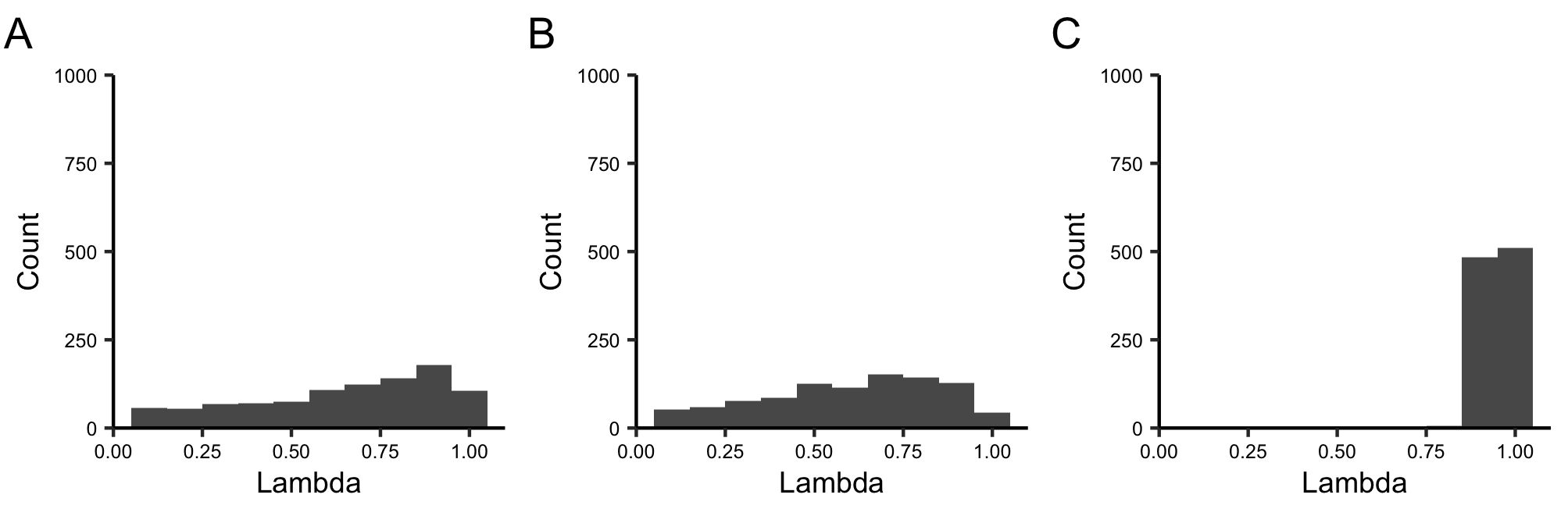
 Figure S2:** Posterior distributions of lambda estimates for CX volume. A) cetaceans only; B) cetaceans only with RoB included as a cofactor; C) all mammals with RoB included as a cofactor.

- 1. **Power to estimate lambda**

To test whether the broad distributions of lambda estimates are due to a lack of power we used the PMC package (Boettiger et al., 2012 Evolution 66(7): 2240-2251) to simulate data across our cetacean-only and all-mammals datasets, with lambda set to either 0.5 and 0.8. Using these data we examined the ability to accurately favour a model in which lambda is estimated (“lambda”) to one where it is set to 1 (“brownian motion”) using PMC’s parametric bootstrapping approach with 1000 bootstraps.

These simulations confirm that there is little power to favour models in which lambda deviates from 1 when phylogenetic signal is moderate (lambda = 0.8) in a phylogenetic dataset structured like our cetacean-only dataset (Table S1; Figure S3).

Given these results, we suggest caution when interpreting the results of models including free estimates of lambda in our PGLS analyses, and favour models in which it is conservatively fixed to 1. In contrast, power is much greater in the all-mammal dataset, and confidence that these estimates are robust should be higher.

**Table S1: Model comparisons using simulated data**

| **Tree** | **Lambda** | **LogLikelihood** | |  |  |  |
| --- | --- | --- | --- | --- | --- | --- |
|  |  | **BM** | **Lambda** | **LR** | **p** |  |
| Ceatceans-only | 0.5 | -58.339 | -52.840 | 10.998 | <0.001 |  |
|  | 0.8 | -52.992 | -52.284 | 1.416 | 0.234 |  |
| All-mammals | 0.5 | -566.609 | -531.255 | 70.708 | <0.001 |  |
|  | 0.8 | -500.686 | -493.481 | 14.410 | <0.001 |  |


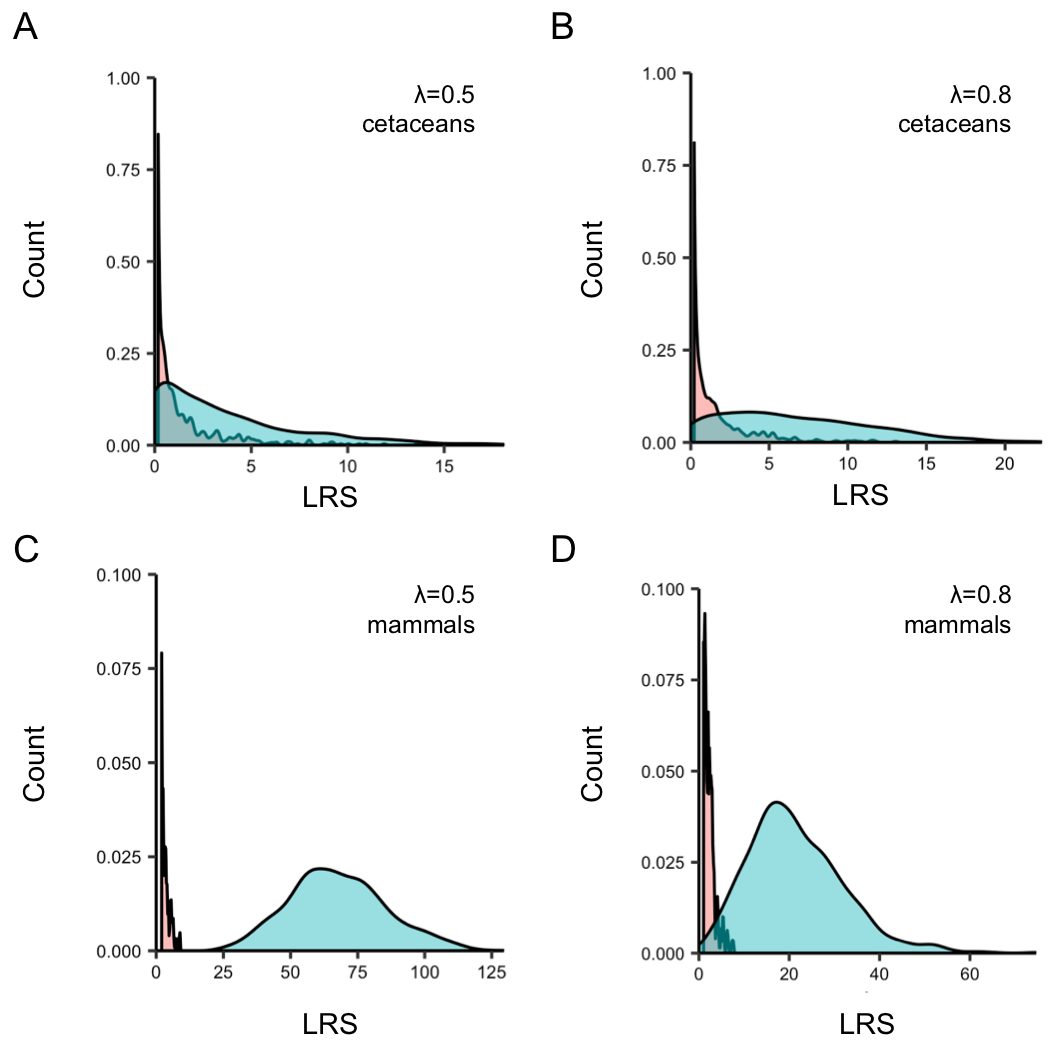


**Figure S3:** Distributions of the likelihood ratio statistic comparing “brownian motion” (red distributions) and “Lambda” (blue distributions) models using data simulated across the cetacean-only (A,B) and all-mammal (C,D) trees, with lambda set to 0.5 (A,C) or 0.8 (C,D) in the simulations.
